# Research on AUV Underwater Localization Method Based on an n-Shaped Array

**DOI:** 10.3390/s26061845

**Published:** 2026-03-15

**Authors:** Chuang Han, Mengran Gao, Tao Shen, Chengli Guo

**Affiliations:** 1 School of Measurement-Control Technology and Communication Engineering, Harbin University of Science and Technology, Harbin 150080, China; hanchuang@hrbust.edu.cn (C.H.); 18332276152@163.com (M.G.); 2 Sunny Group Co., Ltd., Yuyao 315400, China; jtwanghf@sunnyoptical.com

**Keywords:** n-shaped array, MUSIC algorithm, SAGE algorithm, coherent signals, far-field localization, near-field source localization

## Abstract

During continuous navigation of the mother ship, an autonomous underwater vehicle (AUV) can be recovered through an underwater hangar, and the accurate localization of the AUV relative to the mother ship is a key step in the recovery process. To address the AUV localization problem, an n-shaped hydrophone array is designed based on the spatial configuration of the underwater hangar. Since underwater acoustic signals are susceptible to multipath propagation, co-channel interference, and other transmission impairments, the signals received by the array often exhibit coherence. Accordingly, a far-field sound source localization method based on the n-shaped array is proposed. The proposed algorithm first applies spatial smoothing to the *x*-axis and *y*-axis subarrays and jointly constructs a received data vector, followed by eigenvalue decomposition of the corresponding covariance matrix. The Multiple Signal Classification (MUSIC) algorithm is then employed to obtain coarse estimates of the source angles. These coarse estimates are subsequently used as initial values for the Space-Alternating Generalized Expectation-maximization (SAGE) algorithm, which performs refined optimization of the angular parameters in a continuous parameter space, thereby effectively improving estimation accuracy. Furthermore, the proposed algorithm is extended from far-field scenarios to near-field localization. Simulation results demonstrate that the proposed method achieves good parameter estimation performance.

## 1. Introduction

AUVs are essential platforms for underwater exploration and reconnaissance. Common recovery methods for AUVs include ship-based recovery using a support vessel and recovery via fixed platforms. In certain operational scenarios, however, the support vessel is required to recover the AUV while maintaining continuous navigation. Under such conditions, recovery through an underwater docking hanger mounted on the mother ship has received increasing attention. In underwater environments, acoustic waves constitute the primary medium for information transmission. AUVs are typically equipped with acoustic beacons or generate radiated noise during operation, which can be captured by a receiving array. By processing the received acoustic signals, the spatial parameters of the AUV can be estimated. Therefore, the AUV recovery and localization problem in underwater environments can be formulated as an acoustic source localization problem. Source localization is one of the fundamental research topics in the field of array signal processing, and its primary objective is to process the signals received by a sensor array in order to estimate the spatial parameters of the source, such as azimuth, elevation, and range. This framework can be further extended to the estimation of additional parameters, including signal frequency and time delay [1].

The array geometry plays a crucial role in determining the performance of source localization algorithms, and different array configurations demonstrate notable differences in parameter estimation accuracy and implementation complexity. In underwater acoustic source localization studies, commonly employed array structures include the Uniform Linear Array (ULA), the L-shaped array, and the planar array. The ULA is structurally simple and easy to implement; however, it is inherently constrained in multidimensional parameter estimation and cannot provide the unambiguous and accurate joint estimation of multiple spatial parameters such as azimuth, elevation, and range. Although the planar array can offer higher-dimensional spatial information, its structure is more complex and typically requires a larger number of array elements as well as higher deployment precision, which may lead to deployment challenges and increased system costs in practical underwater engineering scenarios. Considering the structural characteristics and spatial layout of the underwater hangar of the mother ship, an n-shaped hydrophone array is deployed near the hangar entrance. This array configuration makes efficient use of the available physical space and provides advantages in multidimensional parameter estimation. The n-shaped array can receive acoustic signals emitted by an acoustic beacon carried by the AUV, as well as radiated noise generated by the vehicle itself. By processing and analyzing the received signals, the spatial parameters of the target sound source, including angular parameters and range, can be accurately estimated, thereby enabling reliable localization of the AUV.

For underwater operational missions of AUVs, localization requirements typically include both direction of arrival (DOA) estimation under long-range conditions and accurate position estimation under short-range conditions. Depending on the distance between the sound source and the receiving array relative to the array aperture, source localization problems are generally classified into far-field and near-field localization. When the sound source is located in the far-field region, the wavefront impinging on the array can be well approximated as a plane wave. In contrast, when the source lies within the Fresnel region of the array, the plane-wave assumption is no longer valid [2]. In this case, the received wavefront exhibits spherical wavefront characteristics, and the phase differences across the array depend on both the source direction and range [3]. Consequently, near-field sound source localization depends not only on the DOA but also on the distance between the source and the reference sensor of the array. For far-field acoustic source localization, commonly used approaches include the Conventional beamforming (CBF), Minimum Variance Distortionless Response (MVDR) [4], and MUSIC algorithms [5]. Among these methods, the MUSIC algorithm exhibits superior estimation performance; however, because it relies on spectral peak search to determine the source direction, it suffers from relatively high computational complexity. To address this issue, a series of improved MUSIC-based methods [6,7,8] as well as the Estimation of Signal Parameters via Rotational Invariance Technique (ESPRIT) [9,10] and the propagator method [11,12,13] have been proposed. For near-field acoustic source localization, several methods have also been developed, including the near-field MVDR algorithm [14], the maximum likelihood (ML) method [15], the near-field MUSIC algorithm [16,17], and the covariance approximation (CA) method [18].

During underwater propagation, acoustic signals are easily affected by multipath propagation and co-channel interference, which often cause the signals received by the array to exhibit coherence. When the incident signals are coherent, the covariance matrix of the array observations becomes rank-deficient. As a result, the dimension of the signal subspace is smaller than the actual number of sound sources, leading to performance degradation or even failure of conventional DOA estimation algorithms. To address DOA estimation under coherent signal conditions, extensive research efforts have been reported in the literature [19,20,21,22]. In [19], a joint covariance matrix was constructed using a spatial smoothing technique, and the ESPRIT algorithm was then applied to estimate the directions of arrival, thereby improving estimation accuracy. In [20], a spatial-smoothing-based ESPRIT was proposed. In this approach, a modified covariance matrix is first obtained through spatial smoothing applied to two parallel linear arrays, and the DOA estimation is subsequently achieved by exploiting the rotational invariance property between subarrays. In [21], the received data vectors of the array elements were used to construct a Toeplitz matrix. A modified covariance matrix was then obtained through Hermitian transpose-based correction and forward–backward processing, and coherent-signal DOA estimation was finally realized by integrating the ESPRIT algorithm.

The methods discussed above mainly focus on DOA estimation for coherent signals in far-field scenarios. In contrast, research on near-field coherent signal localization has also attracted increasing attention [23,24,25,26]. In [23], an efficient iterative algorithm was proposed for the localization of near-field coherent sources. In each iteration, a covariance matrix containing information from only a single source is constructed using the alternating oblique projection (AOP) technique, the DOA is estimated based on the principle of vector inner-product principle, and the corresponding range parameter is finally obtained using one-dimensional ML estimation. In [24], a focusing technique was first applied to approximate the near-field signal model as a far-field model, after which spatial smoothing and ESPRIT were employed for decorrelation and coarse estimation and the AOP was finally iteratively applied to achieve refined estimation. In [25], a planar-array-based approach was proposed for near-field coherent sound source localization. By properly designing the sensor positions, a covariance matrix that does not suffer from rank deficiency under coherent source conditions was constructed. The azimuth angle and range of the sound source were then estimated through two separate one-dimensional searches. In time-reversal (TR) applications, the MUSIC algorithm has also been widely studied [27,28]. In [27], a target localization method based on the multistatic array data matrix was proposed. By performing singular value decomposition (SVD) on the data matrix and combining time-reversal imaging with the MUSIC algorithm, the target position can be estimated. This method demonstrates robustness and high-resolution localization capability in complex propagation environments. In [28], the TR-MUSIC imaging and localization method was investigated, and a theoretical performance analysis model for target position estimation error was established. The root mean square error (RMSE) expression under high signal-to-noise ratio (SNR) conditions was derived, and the influence of noise on localization accuracy was analyzed using SVD perturbation theory.

To provide a clearer overview of existing research, the related literature is summarized and categorized according to several criteria, including array configuration, signal model (far-field or near-field), signal characteristics (coherent or incoherent), and localization algorithms, as shown in Table 1. As shown in Table 1, most existing studies focus on conventional array configurations such as the ULA, the L-shaped array, and planar arrays. In contrast, this paper proposes an n-shaped array based on the structural characteristics of the mother ship’s underwater cabin and investigates far-field and near-field acoustic source localization methods suitable for AUV localization scenarios.

In conventional MUSIC-based localization, a two-dimensional joint search over elevation and azimuth angles is required in far-field scenarios. In near-field cases, an additional range parameter must be introduced, resulting in a three-dimensional search over elevation, azimuth, and range. Due to the strong coupling among these parameters, the achievable scanning resolution is limited, which degrades estimation accuracy. To address the aforementioned issues, this paper proposes an improved MUSIC-based sound source localization algorithm based on an n-shaped array. The proposed algorithm first applies spatial smoothing to the *x*-axis and *y*-axis subarrays and jointly constructs a received data vector, after which an equivalent covariance matrix is formed to effectively restore the dimensionality of the signal subspace in the presence of coherent sources. The MUSIC algorithm is then employed to obtain coarse estimates of the source angles, which are subsequently used as initial values for the SAGE algorithm to perform refined optimization of the angular parameters in a continuous parameter space, thereby effectively improving the estimation accuracy. Simulation results demonstrate that the proposed algorithm achieves good estimation performance.

The structure of this paper is as follows: Section 2 describes the far- field and near-field signal models for the n-shaped array. Section 3 introduces the fundamental principles of the SAGE algorithm and subsequently presents the proposed far- field and near-field acoustic source localization algorithms based on the n-shaped array. Section 4 presents simulation results and analysis of the proposed algorithms. Section 5 concludes the paper.

Symbols: matrices, vectors and scalars are represented by capital bold letters, lower-case bold letters and lowercase letters, respectively. ${\left(\cdot \right)}^{H}$, ${\left(\cdot \right)}^{T}$, ${\left(\cdot \right)}^{*}$ denote conjugate transpose, transpose and conjugate, respectively. E[⋅] represents mathematical expectation. $I_{m}$ and diag(⋅) denote m×m identity matrix and diagonal matrix. $e^{\left(\cdot \right)}$ represents the exponential function, where e denotes Euler’s number.

## 2. Signal Method

During AUV underwater operational missions, the positioning requirements generally involve target direction estimation under long-range conditions and high-precision position estimation under short-range conditions. In long-range scenarios, the primary objective is to estimate the target direction, thereby providing coarse guidance information, while in short-range scenarios, accurate joint estimation of angular and range parameters is required to meet the stringent localization requirements during the recovery phase. These two operational stages correspond to the far-field and near-field acoustic source localization problems, respectively. In long-range scenarios, with respect to the receiving array, the AUV can be reasonably approximated as a far-field acoustic source, and the localization task primarily involves DOA estimation. In contrast, during the short-range recovery stage, the AUV enters the near-field region of the array, where joint estimation of angular and range parameters is required for precise localization. Therefore, simultaneous investigation of far-field and near-field models is essential for establishing a unified theoretical framework capable of meeting the full-process localization requirements of AUV operations.

**Figure 1:**
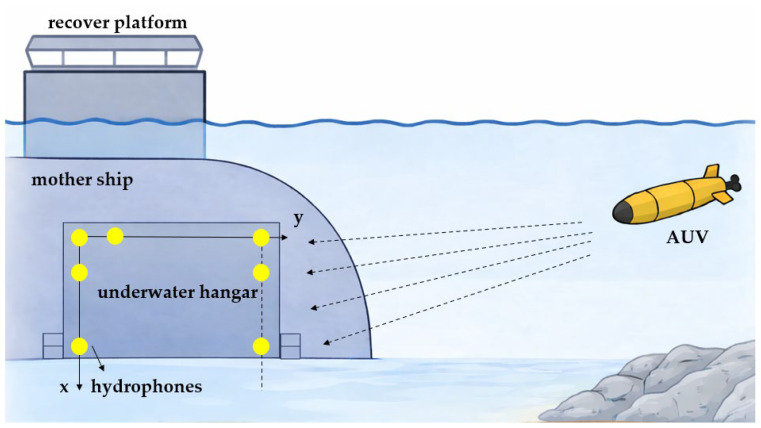
Scenario diagram.

Due to the structural characteristics of the underwater hangar and the fact that hydrophones are typically installed on the inner side of the cabin wall, the array geometry must be compatible with the planar structure of the cabin wall as well as the limited available installation space. The n-shaped array configuration proposed in this paper allows array elements to be arranged along two orthogonal directions of the cabin wall, thereby preserving a relatively large array aperture while satisfying practical installation constraints. In addition, this configuration facilitates the cable routing and mechanical mounting of the hydrophones, making it suitable for space-constrained underwater cabin environments. The scenario is illustrated in Figure 1.

**Figure 2:**
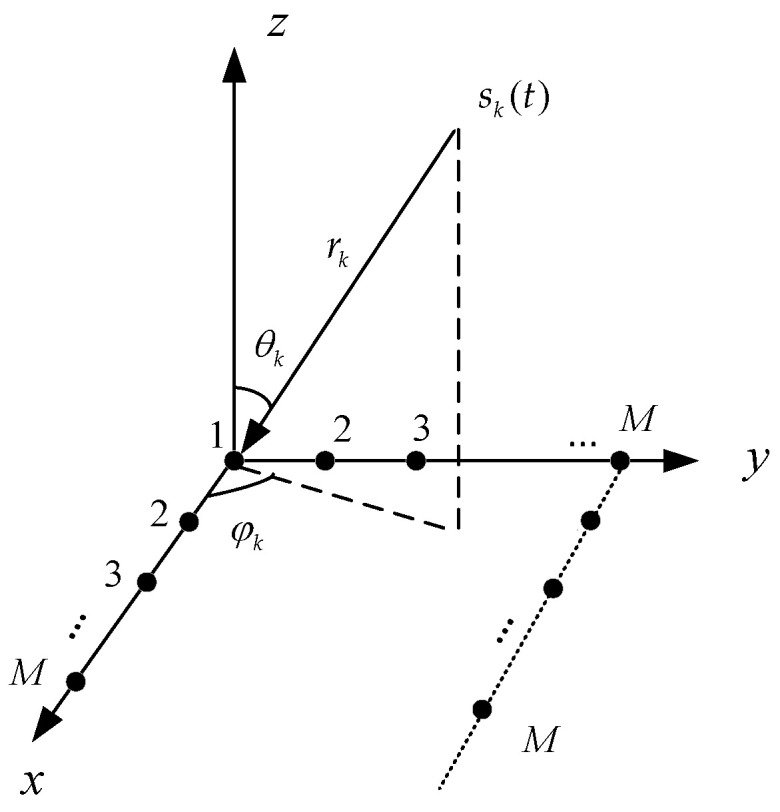
n-shape array structure.

The structure of the n-shaped array is illustrated in Figure 2, which consists of three uniform linear arrays arranged in the *x–y* plane along the *x*-axis, the *y*-axis, and a direction parallel to the *x*-axis, respectively, with the sensor at the coordinate origin serving as the central reference element. Each subarray is composed of *M* sensors, resulting in a total of 3*M−*2 array elements. The inter-element spacing is denoted by *d*, and the distance between the subarray along the *x*-axis and the subarray parallel to the *x*-axis is denoted by l=(M-1)d. It is assumed that *K* narrowband signals impinge on the n-shaped array, and the number of sources *K* is known a priori in this paper, where the directional information of the *k*-th incident signal is characterized by the pair of angles $\left(\theta_{k},\varphi_{k}\right)$, Here, $\theta_{k}$ and $\varphi_{k}$ denote the elevation angle and the azimuth angle, respectively, corresponding to the angle between the incident signal and the positive *z*-axis, and the angle between the projection of the incident signal onto the *x–y* plane and the positive *x*-axis. The distance from the k-th source to the reference array element is denoted by $r_{k}$.

The received data vectors of the three subarrays located along the *x*-axis, the *y*-axis, and the direction parallel to the *x*-axis can be expressed as

(1) $$X_{1}(t)=A_{x1}S(t)+N_{x1}(t),$$

(2) $$Y\left(t\right)=A_{y}S(t)+N_{y}(t),$$

(3) $$X_{2}(t)=A_{x2}S(t)+N_{x2}(t).$$

where $S(t)={\left[s_{1}(t),s_{2}(t),s_{3}(t),\ldots ,s_{K}(t)\right]}^{T}$ denotes the incident signal vector. The noise vector of the *x*-axis subarray is denoted by $N_{x1}(t)={\left[n_{x11}(t),n_{x12}(t),\ldots ,n_{x1M}(t)\right]}^{T}$, that of the *y*-axis subarray is denoted by $N_{y}(t)={\left[n_{y1}(t),n_{y2}(t),\ldots ,n_{yM}(t)\right]}^{T}$, and the noise vector of the subarray parallel to the *x*-axis is denoted by $N_{x2}(t)={\left[n_{x21}(t),n_{x22}(t),\ldots ,n_{x2M}(t)\right]}^{T}$; all noise vectors are assumed to be zero-mean Gaussian white noise with variance $\sigma^{2}$.

As shown in Figure 2, it is assumed that *K* far-field narrowband signals impinge on the n-shaped array. The array steering vector matrices corresponding to the subarrays along the *x*-axis, the *y*-axis, and the subarray parallel to the *x*-axis are denoted by $A_{x1}$, $A_{y}$, and $A_{x2}$, respectively, and can be expressed as follows:

(4) $$A_{x1}=\left[a_{x1}(\theta_{1},\varphi_{1}),a_{x1}(\theta_{2},\varphi_{2}),\ldots a_{x1}(\theta_{K},\varphi_{K})\right],$$

(5) $$A_{y}=\left[a_{y}(\theta_{1},\varphi_{1}),a_{y}(\theta_{2},\varphi_{2}),\ldots a_{y}(\theta_{K},\varphi_{K})\right],$$

(6) $$A_{x2}=\left[a_{x2}(\theta_{1},\varphi_{1}),a_{x2}(\theta_{2},\varphi_{2}),\ldots a_{x2}(\theta_{K},\varphi_{K})\right]=A_{x1}\Phi .$$

where

(7) $$a_{x1}(\theta_{k},\varphi_{k})={\left[1,e^{-j2\pi d\cos\varphi_{k}\sin\theta_{k}/\lambda },\ldots ,e^{-j2\pi (M-1)d\cos\varphi_{k}\sin\theta_{k}/\lambda }\right]}^{T},$$

(8) $$a_{y}(\theta_{k},\varphi_{k})={\left[1,e^{-j2\pi d\sin\varphi_{k}\sin\theta_{k}/\lambda },\ldots ,e^{-j2\pi (M-1)d\sin\varphi_{k}\sin\theta_{k}/\lambda }\right]}^{T},$$

(9) $$\Phi =diag(e^{-j2\pi l\sin\varphi_{1}\sin\theta_{1}/\lambda },e^{-j2\pi l\sin\varphi_{2}\sin\theta_{2}/\lambda },\ldots ,e^{-j2\pi l\sin\varphi_{K}\sin\theta_{K}/\lambda }).$$

In the near-field scenario, the array steering vector matrices corresponding to the subarrays along the *x*-axis, the *y*-axis, and the subarray parallel to the *x*-axis are denoted by $A_{x1}$, $A_{y}$, and $A_{x2}$, respectively, and can be expressed as follows:

(10) $$A_{x1}=\left[a_{x1}(\theta_{1},\varphi_{1},r_{1}),a_{x1}(\theta_{2},\varphi_{2},r_{2}),\ldots a_{x1}(\theta_{K},\varphi_{K},r_{K})\right],$$

(11) $$A_{y}=\left[a_{y}(\theta_{1},\varphi_{1},r_{1}),a_{y}(\theta_{2},\varphi_{2},r_{2}),\ldots a_{y}(\theta_{K},\varphi_{K},r_{K})\right],$$

(12) $$A_{x2}=\left[a_{x2}(\theta_{1},\varphi_{1},r_{1}),a_{x2}(\theta_{2},\varphi_{2},r_{2}),\ldots a_{x2}(\theta_{K},\varphi_{K},r_{K})\right].$$

The steering vectors corresponding to the x-axis subarray, the y-axis subarray, and the subarray parallel to the x-axis are given, respectively, as follows:

(13) $$a_{x1}(\theta_{k},\varphi_{k},r_{k})={\left[1,e^{j(\gamma_{xk}+\phi_{xk})},\ldots ,e^{j[\gamma_{xk}(M-1)+\phi_{xk}{\left(M-1\right)}^{2}]}\right]}^{T},$$

(14) $$a_{y}(\theta_{k},\varphi_{k},r_{k})={\left[1,e^{j(\gamma_{yk}+\phi_{yk})},\ldots ,e^{j[\gamma_{yk}(M-1)+\phi_{yk}{\left(M-1\right)}^{2}]}\right]}^{T},$$

(15) $$a_{x2}(\theta_{k},\varphi_{k},r_{k})=\left[\begin{array}{c} e^{j(\gamma_{yk}b+\phi_{yk}b^{2})}\\ e^{j(\gamma_{xk}+\phi_{xk}+\gamma_{yk}b+\phi_{yk}b^{2}+\beta_{k}b)}\\ \vdots \\ e^{j[\gamma_{xk}(M-1)+\phi_{xk}{\left(M-1\right)}^{2}+\gamma_{yk}b+\phi_{yk}b^{2}+\beta_{k}(M-1)b]} \end{array}\right].$$

where k=1,2,…,K, b=M−1

(16) $$\gamma_{xk}=-2\pi \frac{d}{\lambda }\sin\theta_{k}\cos\varphi_{k},\phi_{xk}=\frac{\pi d^{2}}{\lambda r_{k}}(1-\sin^{2}\theta_{k}\cos^{2}\varphi_{k}).$$

(17) $$\gamma_{yk}=-2\pi \frac{d}{\lambda }\sin\theta_{k}\sin\varphi_{k},\phi_{yk}=\frac{\pi d^{2}}{\lambda r_{k}}(1-\sin^{2}\theta_{k}\sin^{2}\varphi_{k}).$$

(18) $$\beta_{k}=-\pi \frac{d^{2}}{\lambda r_{k}}\sin^{2}\theta_{k}\sin2\varphi_{k}.$$

## 3. Algorithm Principle

To address the requirements of long-range orientation and short-range precise localization in the AUV positioning process, this paper proposes a far-field acoustic source localization algorithm and a near-field acoustic source localization algorithm based on the n-shaped array, respectively. This section systematically presents the theoretical foundations and key steps of the two algorithms.

### 3.1. SAGE Algorithm

The SAGE algorithm reformulates the joint multi-source estimation problem as an alternating estimation process performed for individual sources. Its core principle is to update only a subset of parameters associated with the current source during each iteration while keeping the parameters of the remaining sources fixed [29,30]. By adopting this space-alternating strategy, the algorithm reduces computational complexity and enhances convergence stability.

Assume that the signal received by the array is given by

(19) $$X(t)=\sum_{k=1}^{K}a(\theta_{k})s_{k}(t)+N(t).$$

where $a(\theta_{k})$ denotes the steering vector corresponding to the *k*-th signal, $s_{k}(t)$ represents the complex envelope of the *k*-th signal, and N(t) is additive white Gaussian noise with zero mean and variance $\sigma^{2}$.

Given that the number of sources *K* is known, the goal is to estimate the desired parameters from the observation data X(t). The complete data corresponding to the *k*-th signal is defined as

(20) $$X_{k}(t)=a(\theta_{k})s_{k}(t)+N(t).$$

The SAGE algorithm consists of two steps: the expectation step (E-step) and the maximization step (M-step).

1. E-step

The core assumption of the SAGE algorithm is that, if the estimates of the other j≠k sources are known, the expected observation corresponding to the *k*-th source can be reconstructed.

Based on conditional probability, and given the observation data X(t) and the current parameter estimates $\hat{\theta }$, the complete data estimate corresponding to the *k*-th source is obtained:

(21) $$\begin{array}{cc} {\hat{X}}_{k}^{\left(i\right)}(t)= & E[X_{k}(t)|X(t),{\hat{\theta }}^{\left(i-1\right)}]\\ & =a({\hat{\theta }}_{k}^{\left(i-1\right)}){\hat{s}}_{k}^{\left(i-1\right)}(t)+(X(t)-\sum_{j=1}^{K}a({\hat{\theta }}_{j}^{\left(i-1\right)}){\hat{s}}_{j}^{\left(i-1\right)}(t)) \end{array}.$$

Under the Gaussian white noise assumption, the following approximation is commonly adopted:

(22) $${\hat{X}}_{k}^{\left(i\right)}(t)=X(t)-\sum_{j\neq k}a({\hat{\theta }}_{j}^{\left(i-1\right)}){\hat{s}}_{j}^{\left(i-1\right)}(t).$$

where $a({\hat{\theta }}_{k}){\hat{s}}_{k}$ denotes the current reconstruction of the *k*-th signal component, and *i* denotes the iteration index. Let i=0 denote the initialization stage. The initial angle estimates ${\hat{\theta }}_{k}^{\left(0\right)}$ are obtained from the MUSIC spectrum, and the corresponding source signals are initialized via least-squares projection. At iteration i≥1, the E-step uses the estimates from iteration i−1, and the M-step updates the parameters to obtain the *i*-th estimates. The iterations continue until the relative parameter variation falls below a predefined threshold or the maximum iteration number is reached.

2. M-step

After obtaining the estimate ${\hat{X}}_{k}(t)$, the parameters of the *k*-th source, $\theta_{k}$ and $s_{k}$, are updated by maximizing the conditional expected log-likelihood function. Under the additive white Gaussian noise assumption, the parameter update problem can be reformulated as a least-squares optimization:

(23) $$\{{\hat{\theta }}_{k}^{\left(i\right)},{\hat{s}}_{k}^{\left(i\right)}\}=\arg\min_{\theta_{k}}\sum_{t=1}^{T}\|{\hat{X}}_{k}^{\left(i\right)}(t)-a(\theta_{k})s_{k}(t){\|}^{2}.$$

Assuming that ${\hat{\theta }}_{k}$ is known, taking the partial derivative of (23) with respect to $s_{k}$ yields

(24) $${\hat{s}}_{k}^{\left(i\right)}(t)=\frac{a^{H}(\theta_{k}){\hat{X}}_{k}^{\left(i\right)}(t)}{a^{H}(\theta_{k})a(\theta_{k})}.$$

By substituting (24) into (23), the problem can be reformulated as maximizing the spatial projection power:

(25) $${\hat{\theta }}_{k}^{\left(i\right)}=\arg\max_{\theta_{k}}\frac{\|a^{H}(\theta_{k}){\hat{X}}_{k}^{\left(i\right)}(t){\|}^{2}}{a^{H}(\theta_{k})a(\theta_{k})}.$$

For a uniform linear array, $a{\left(\theta_{k}\right)}^{H}a(\theta_{k})=M$, (25) can be simplified as follows:

(26) $${\hat{\theta }}_{k}^{\left(i\right)}=\arg\max_{\theta_{k}}{\left|a^{H}(\theta_{k}){\hat{X}}_{k}^{\left(i\right)}(t)\right|}^{2}.$$

### 3.2. Far-Field Sound Source Location Algorithm

Due to multipath propagation and other effects in the underwater environment, the signals received by an array often exhibit coherence, under which the covariance matrix of the received data becomes rank-deficient. In this case, the subspace orthogonality property on which the conventional MUSIC algorithm relies no longer holds, making it difficult to achieve effective parameter estimation directly. To address the rank-deficiency problem, the covariance matrix of the received data can be preprocessed using spatial smoothing techniques to restore its full-rank property, after which the MUSIC algorithm can be applied to perform sound source localization.

The spatial smoothing algorithm requires the array structure to satisfy translational invariance. For the n-shaped array, both the subarrays along the *x*-axis and the *y*-axis possess translational invariance and can therefore be processed using spatial smoothing. However, for the subarray parallel to the *x*-axis, the inter-element phase differences include a term related to lsinθsinφ, where l=(M−1)d is a constant, which violates the translational invariance between subarrays and thus precludes the direct application of spatial smoothing. Consequently, for DOA estimation of coherent far-field signals based on the n-shaped array, spatial smoothing can first be applied to the *x*-axis and *y*-axis subarrays to mitigate signal coherence and restore the full-rank property of the covariance matrix, after which the azimuth and elevation angles of the sound sources can be estimated using the MUSIC algorithm.

The arrays along the *x*- and *y*-axes are each partitioned into *L* subarrays, with each subarray consisting of q=M−L+1 array elements. Then, the received data from the *p*-th subarrays along the *x*-axis and the *y*-axis can be expressed, respectively, as follows:

(27) x1,pf(t)=x1,p(t),x1,p+1(t),…,x1,p+q−1(t)T=Ax1,qDx1s(t)p−1+nxp(t).

(28) ypf(t)=yp(t),yp+1(t),…,yp+q−1(t)T=Ay,qDy1s(t)p−1+nyp(t).

where $A_{x1,q}$ and $A_{y,q}$ represent the first *q* rows of the steering vector matrices $A_{x1}$ and $A_{y}$ associated with the *x*-axis and *y*-axis subarrays, i.e., the first *q* rows of (4) and (5), respectively; and $D_{x1}$ $D_{y1}$ are diagonal matrices given by

(29) $$D_{x1}=\left[\begin{array}{cccc} e^{-j2\pi d\cos\varphi_{1}\sin\theta_{1}/\lambda } & 0 & \ldots & 0\\ 0 & e^{-j2\pi d\cos\varphi_{2}\sin\theta_{2}/\lambda } & \ldots & 0\\ \vdots & \vdots & \ddots & \vdots \\ 0 & 0 & \ldots & e^{-j2\pi d\cos\varphi_{K}\sin\theta_{K}/\lambda } \end{array}\right].$$

(30) $$D_{y}=\left[\begin{array}{cccc} e^{-j2\pi d\sin\varphi_{1}\sin\theta_{1}/\lambda } & 0 & \ldots & 0\\ 0 & e^{-j2\pi d\sin\varphi_{2}\sin\theta_{2}/\lambda } & \ldots & 0\\ \vdots & \vdots & \ddots & \vdots \\ 0 & 0 & \ldots & e^{-j2\pi d\sin\varphi_{K}\sin\theta_{K}/\lambda } \end{array}\right].$$

A received data vector jointly formed by the *p*-th forward spatially smoothed subarrays of the *x*-axis and *y*-axis is constructed, whose expression can be written as

(31) $$z_{p}(t)=\left[\begin{array}{l} x_{1,p}^{f}(t)\\ y_{p}^{f}(t) \end{array}\right].$$

The corresponding steering vector of the joint subarray can be expressed as

(32) $$a_{z}(\theta ,\varphi )=\left[\begin{array}{l} a_{x1}(\theta ,\varphi )\\ a_{y}(\theta ,\varphi ) \end{array}\right].$$

By averaging the covariance matrices of the *L* subarrays, the forward spatially smoothed covariance matrix $R_{z}^{f}$ is obtained

(33) $$R_{z}^{f}=\frac{1}{L}\frac{1}{N}\sum_{p=1}^{L}\sum_{t=1}^{N}z_{p}^{f}(t)z_{p}^{fH}(t).$$

The forward–backward spatially smoothed covariance matrix can then be further derived

(34) $$R_{z}^{fb}=\frac{1}{2}(R_{z}^{f}+J{\left(R_{z}^{f}\right)}^{*}J).$$

By performing eigenvalue decomposition on the covariance matrix, the signal subspace $U_{S}$ and the noise subspace $U_{N}$ can be obtained

(35) $$R_{z}^{fb}=U_{S}\Lambda_{S}U_{S}^{H}+U_{N}\Lambda_{N}U_{N}^{H}.$$

Therefore, the spatial spectrum function can be obtained as follows:

(36) $$P_{MUSIC}(\theta ,\varphi )=\frac{1}{a_{z}^{H}(\theta ,\varphi )U_{N}U_{N}^{H}a_{z}(\theta ,\varphi )}.$$

Under far-field conditions, the MUSIC algorithm based on the n-shaped array requires a two-dimensional joint search over the azimuth and elevation angles. Owing to the strong coupling between these two parameters, the achievable search resolution and estimation accuracy are limited. To overcome this limitation, an improved MUSIC algorithm is proposed. First, coarse estimates of the source angles are obtained using the MUSIC algorithm, and these estimates are then used as initial values for refined estimation of the angular parameters in a continuous parameter space via the SAGE algorithm, thereby effectively improving the estimation accuracy of two-dimensional far-field DOA estimation.

Coarse estimates of the source angles are obtained by performing peak searching on the spatial spectrum

(37) $$\left({\hat{\theta }}_{k}^{\left(0\right)},{\hat{\varphi }}_{k}^{\left(0\right)}\right)=\arg\max P_{MUSIC}(\theta ,\varphi ).$$

where $\left({\hat{\theta }}_{k}^{\left(0\right)},{\hat{\varphi }}_{k}^{\left(0\right)}\right)$ denotes the coarse estimation results, which can be used as the initial values for the SAGE algorithm.

By subtracting the contributions of all sources except the *k*-th source from the original array observations, the equivalent observation corresponding to the *k*-th source can be obtained

(38) $${\hat{X}}_{nk}(t)=X_{n}(t)-\sum_{j\neq k}a({\hat{\theta }}_{j},{\hat{\varphi }}_{j}){\hat{s}}_{j}(t).$$

The signal waveform is subsequently updated

(39) $${\hat{s}}_{k}(t)=\frac{a^{H}(\theta_{k},\varphi_{k}){\hat{X}}_{nk}(t)}{a^{H}(\theta_{k},\varphi_{k})a(\theta_{k},\varphi_{k})}.$$

The angular parameters are updated based on the maximum likelihood criterion ${\hat{\theta }}_{k}$, ${\hat{\varphi }}_{k}$

(40) $$({\hat{\theta }}_{k},{\hat{\varphi }}_{k})=\arg\max_{\theta_{k},\varphi_{k}}{\left|a^{H}(\theta_{k},\varphi_{k}){\hat{X}}_{nk}(t)\right|}^{2}.$$

Let the maximum number of iterations be Q and the allowable estimation error be ε, the iteration process terminates when the iteration count reaches Q or when the condition in (41) is satisfied

(41) $$\|{\hat{\Theta }}_{1}^{\left(i\right)}-{\hat{\Theta }}_{1}^{\left(i-1\right)}\|\leq \varepsilon .$$

where $\Theta_{1}=\left[\begin{array}{cc} \theta_{1} & \varphi_{1}\\ \theta_{2} & \varphi_{2}\\ \vdots & \vdots \\ \theta_{K} & \varphi_{K} \end{array}\right]$.

For coherent signals, the procedure of the proposed improved MUSIC-based far-field sound source localization algorithm is summarized as follows:

1. Construct the received data vector $z_{p}(t)$ by jointly combining the forward spatially smoothed subarrays of the *x*-axis and *y*-axis subarrays, compute the forward–backward spatially smoothed covariance matrix $R_{z}^{fb}$ of the joint subarray, and perform eigenvalue decomposition to obtain the noise subspace $U_{N}$;
2. The spatial spectrum function $P_{MUSIC}(\theta ,\varphi )$ is constructed based on the orthogonality between the steering vector $a_{z}(\theta ,\varphi )$ and the noise subspace $U_{N}$.
3. Coarse estimates of the source elevation and azimuth angles $\left({\hat{\theta }}_{k}^{\left(0\right)},{\hat{\varphi }}_{k}^{\left(0\right)}\right)$ are obtained through two-dimensional spectral peak searching and are then used as the initial values for the SAGE algorithm.
4. The equivalent observation signal ${\hat{X}}_{nk}$ of the *k*-th source is constructed according to (38);
5. The signal waveform parameters are updated, followed by updating the angular parameters ${\hat{\theta }}_{k}$ and ${\hat{\varphi }}_{k}$ based on the maximum likelihood criterion;
6. The iteration is terminated when the maximum number of iterations Q is reached or when the condition in (41) is satisfied, and the final estimates of θ and φ are output; otherwise, the algorithm returns to the step 4.

**Figure 3:**
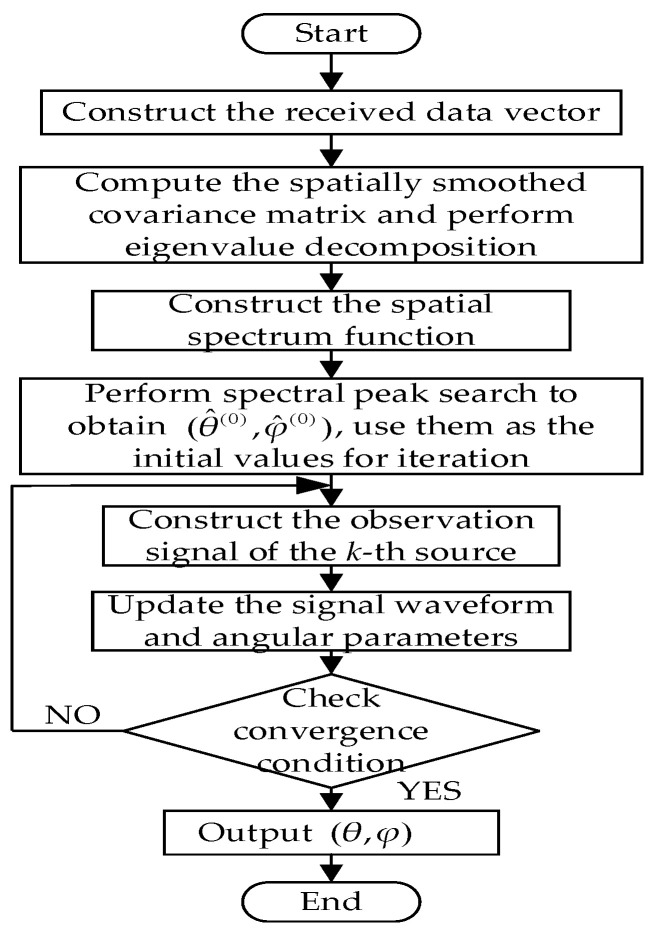
Flowchart of the improved MUSIC far-field sound source localization algorithm.

The flowchart of the algorithm is shown in Figure 3.

### 3.3. Near-Field Sound Source Location Algorithm

The above far-field sound source localization algorithm is extended to the near-field case. As discussed in Section 3.1, the subarray parallel to the *x*-axis does not satisfy translational invariance; therefore, a received data vector is constructed by jointly combining the *p*-th forward spatially smoothed subarrays of the *x*-axis and *y*-axis.

(42) $$z_{p}(t)=\left[\begin{array}{l} x_{1,p}^{f}(t)\\ y_{p}^{f}(t) \end{array}\right].$$

The corresponding steering vector of the joint subarray can be expressed as

(43) $$a_{z}(\theta ,\varphi ,r)=\left[\begin{array}{l} a_{x1}(\theta ,\varphi ,r)\\ a_{y}(\theta ,\varphi ,r) \end{array}\right].$$

By averaging the covariance matrices of the *L* subarrays, the forward spatially smoothed covariance matrix $R_{z}^{f}$ is obtained

(44) $$R_{z}^{f}=\frac{1}{L}\frac{1}{N}\sum_{p=1}^{L}\sum_{t=1}^{N}z_{p}^{f}(t)z_{p}^{fH}(t).$$

The forward–backward spatially smoothed covariance matrix can then be further derived

(45) $$R_{z}^{fb}=\frac{1}{2}(R_{z}^{f}+J{\left(R_{z}^{f}\right)}^{*}J).$$

By performing eigenvalue decomposition on the covariance matrix, the signal subspace $U_{S}$ and the noise subspace $U_{N}$ can be obtained

(46) $$R_{z}^{fb}=U_{S}\Lambda_{S}U_{S}^{H}+U_{N}\Lambda_{N}U_{N}^{H}.$$

Therefore, the spatial spectrum function can be obtained as follows:

(47) $$P_{MUSIC}(\theta ,\varphi ,r)=\frac{1}{a_{z}^{H}(\theta ,\varphi ,r)U_{N}U_{N}^{H}a_{z}(\theta ,\varphi ,r)}.$$

From Equation (36), it can be seen that under near-field conditions, the MUSIC algorithm requires a three-dimensional search over the elevation, azimuth, and range parameters, resulting in a relatively high computational burden. Moreover, owing to the pronounced nonlinear coupling between the angular and range parameters in the near-field array model, different combinations of angle and range may produce similar phase responses; when a direct three-dimensional joint parameter search is performed, the spatial spectrum is prone to exhibiting spurious peaks, thereby degrading the accuracy of parameter estimation. To reduce parameter coupling, the range parameter can be fixed, transforming the original three-dimensional search problem into a two-dimensional search, which effectively reduces the computational complexity while maintaining reliable angle estimation performance.

By fixing the range parameter $r=r_{0}$, $r_{0}$ is a temporarily assumed value, based on which a new spatial spectrum function is constructed according to the orthogonality between the steering vector and the noise subspace

(48) $$P_{MUSIC}(\theta ,\varphi )=\frac{1}{a_{z}^{H}(\theta ,\varphi ,r_{0})U_{N}U_{N}^{H}a_{z}(\theta ,\varphi ,r_{0})}.$$

Coarse estimates of the source angles $\left({\hat{\theta }}_{k}^{\left(0\right)},{\hat{\varphi }}_{k}^{\left(0\right)}\right)$ are obtained through a two-dimensional spectral peak search, and the resulting coarse estimates $\left({\hat{\theta }}_{k}^{\left(0\right)},{\hat{\varphi }}_{k}^{\left(0\right)}\right)$ together with $r=r_{0}$ are used as the initial values for the iterative SAGE algorithm. The SAGE algorithm is then employed to obtain refined estimates of both the angles and the range (θ,φ,r). The iteration is terminated when the maximum number of iterations Q is reached or when the stopping condition in (49) is satisfied.

(49) $$\|{\hat{\eta }}^{\left(i\right)}-{\hat{\eta }}^{\left(i-1\right)}\|\leq \varepsilon .$$

where $\eta =\left[\begin{array}{ccc} \theta_{1} & \varphi_{1} & r_{1}\\ \theta_{2} & \varphi_{2} & r_{2}\\ \vdots & \vdots & \vdots \\ \theta_{K} & \varphi_{K} & r_{K} \end{array}\right]$.

For coherent signals, the procedure of the proposed improved MUSIC-based near-field sound source localization algorithm is summarized as follows:

1. Construct the received data vector $z_{p}(t)$ by jointly combining the forward spatially smoothed subarrays of the *x*-axis and *y*-axis subarrays, compute the forward–backward spatially smoothed covariance matrix $R_{z}^{fb}$ of the joint subarray, and perform eigenvalue decomposition to obtain the noise subspace $U_{N}$.
2. Construct the spatial spectrum function based on the orthogonality between the steering vector $a_{z}(\theta ,\varphi )$ and the noise subspace $U_{N}$, and fix the range parameter $r=r_{0}$ to obtain a modified spatial spectrum function $P_{MUSIC}(\theta ,\varphi )$.
3. Obtain coarse estimates of the source elevation and azimuth angles $\left({\hat{\theta }}_{k}^{\left(0\right)},{\hat{\varphi }}_{k}^{\left(0\right)}\right)$ through two-dimensional spectral peak searching, and these estimates together with the fixed range parameter $r=r_{0}$ are used as the initial values for the SAGE algorithm.
4. Construct the equivalent observation signal ${\hat{X}}_{nk}$ of the *k*-th source.
5. Update the signal waveform ${\hat{s}}_{k}(t)$, and then update the angular and range parameters ${\hat{\theta }}_{k}$ and ${\hat{\varphi }}_{k}$ based on the maximum likelihood criterion.
6. Terminate the iteration when the maximum number of iterations Q is reached or when the condition in (49) is satisfied, and obtain the final estimates θ and φ; otherwise, the algorithm returns to the step 4.

**Figure 4:**
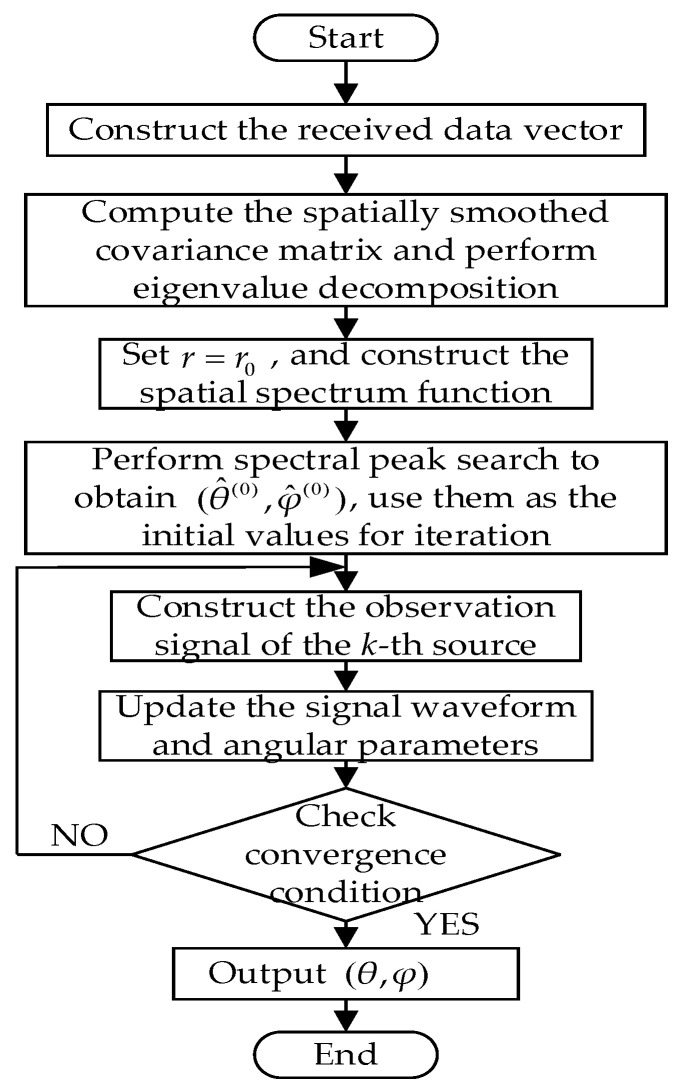
Flowchart of the improved MUSIC near-field sound source localization algorithm.

The flowchart of the algorithm is shown in Figure 4.

The computational complexity of the proposed far-field and near-field acoustic source localization algorithms mainly comprises two stages: the FBSS-MUSIC initialization stage and the SAGE-based refinement stage. Compared with the far-field localization algorithm, the steering vector of the near-field n-shaped array contains a quadratic phase term; however, the complexity of generating the steering vector in a single evaluation remains O(M), and therefore does not change the dominant computational complexity order. In addition, during the initialization stage of the near-field localization algorithm, no discrete search is performed with respect to the range parameter r; instead, the spatial spectrum is evaluated only over the two-dimensional angular domain. Consequently, the computational complexity of the spatial spectrum search in the initialization stage is the same for both the far-field and near-field algorithms. In summary, the computational complexities of the far-field and near-field source localization algorithms are of the same order.

The complexity analysis includes the construction of the spatially smoothed covariance matrix, eigenvalue decomposition, two-dimensional spatial spectrum search, and the iterative parameter optimization process involved in the SAGE algorithm. In the initialization stage, the complexity of constructing the spatially smoothed covariance matrix is $O(Lq^{2}N)$, the complexity of eigenvalue decomposition is $O(q^{3})$, and the complexity of the two-dimensional spatial spectrum search is $O(G_{\theta }G_{\varphi }q^{2})$. In the refinement stage, considering *K* sources and a maximum of Q iterations, the resulting computational complexity is $O(QMK^{2}N)$ Therefore, the overall computational complexity of the proposed algorithm can be summarized as follows:

(50) $$O(Lq^{2}N+q^{3}+G_{\phi }G_{\theta }q^{2}+QMK^{2}N)$$

where *L* denotes the number of spatial smoothing subarrays, q=M−L+1 represents the length of each spatial smoothing subarray, *M* is the number of array elements in the subarray of the n-shaped array, and *N* denotes the number of snapshots. $G_{\theta }=181/\Delta \theta$ represents the number of elevation angle search points, and Δθ is the elevation angle search step size. $G_{\varphi }=181/\Delta \varphi$ denotes the number of azimuth angle search points, and Δφ is the azimuth angle search step size.

## 4. Simulation and Analysis

**Figure 5:**
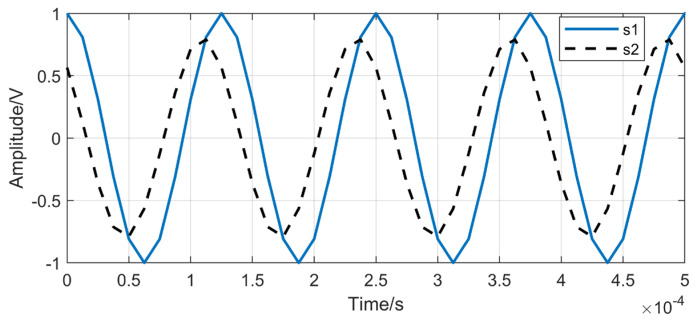
Time-domain waveforms of $s_{1}$ and $s_{2}$.

Assume that two narrowband coherent acoustic sources located in the far-field region, denoted by $s_{1}$ and $s_{2}$, with $s_{2}=0.8e^{-j\pi /4}s_{1}$, both with a frequency of 8 kHz and an amplitude of 1V, and the sampling frequency is set to 80 kHz. The time-domain waveforms of $s_{1}$ and $s_{2}$ are illustrated in Figure 5. In the simulation, additive white Gaussian noise is added at different SNR levels, where the SNR is defined as the ratio of the signal power to the noise power, and its expression is given as follows:

(51) $$SNR=10\log_{10}(P_{S}/P_{N}).$$

where the signal power and noise power are denoted by $P_{S}$ and $P_{N}$, respectively.

In this paper, the RMSE is adopted as a performance metric to evaluate the estimation accuracy of the proposed algorithm. The RMSE of the localization parameters is defined as follows:

(52) $$RMSE(\mu )=\sqrt{\frac{1}{TK}\sum_{j=1}^{T}\sum_{k=1}^{K}{\left({\hat{\mu }}_{kj}-\mu_{k}\right)}^{2}}.$$

where *T* denotes the number of Monte Carlo trials, ${\hat{\mu }}_{kj}$ represents the estimated value of the localization parameter of the *k*-th source obtained in the *j*-th Monte Carlo trial, and $\mu_{k}$ denotes the true value of the localization parameter of the *k*-th source.

### 4.1. Effectiveness Analysis

Simulation Experiment 1: For coherent signals, the effectiveness of the proposed improved MUSIC-based far-field sound source localization algorithm using an n-shaped array is validated.

**Figure 6:**
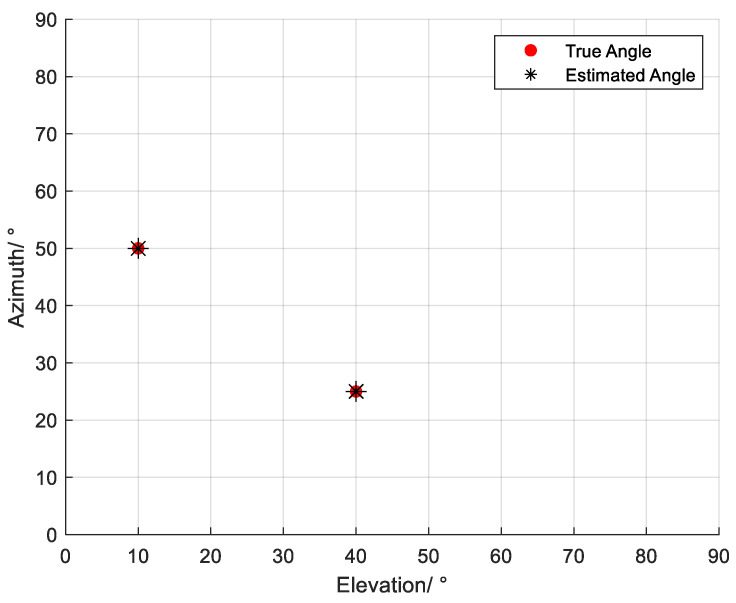
Estimation results of the far-field acoustic source localization algorithm for *K* = 2.

Two narrowband coherent acoustic impinge on the n-shaped array, denoted as $s_{1}$ by $s_{2}$. The incident angles are represented by (θ,φ), where θ denotes the elevation angle and φ denotes the azimuth angle. The incident directions of the two sources are (10°,50°) and (40°,25°), respectively. The numbers of array elements along the *x*-axis, the *y*-axis, and the subarray parallel to the *x*-axis in the n-shaped array are all set to M=7. The sound speed is denoted by c=1500 m/s, and the inter-element spacing is d=λ/2. SNR=20 dB, and the number of snapshots is N=500. The estimation results are shown in Figure 6. It can be observed that the estimated angles agree well with the true values, demonstrating that the proposed improved MUSIC-based far-field sound source localization algorithm based on the n-shaped array is effective and achieves accurate estimation performance.

**Figure 7:**
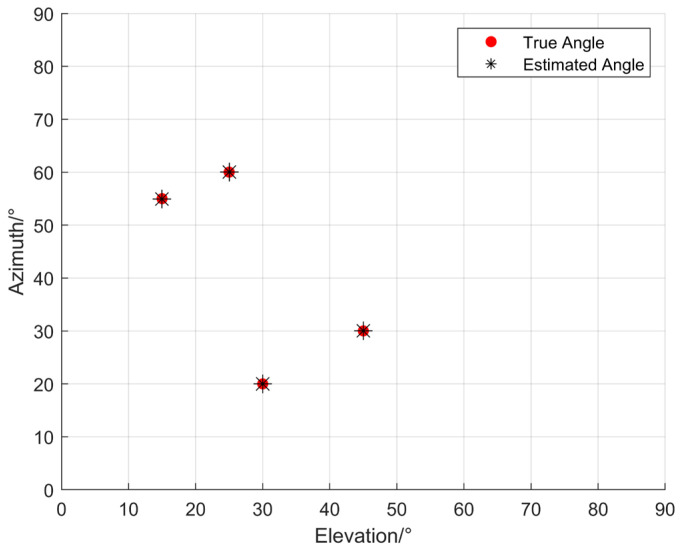
Estimation results of the far-field acoustic source localization algorithm for *K* = 4.

When K=4 narrowband coherent sources impinge on the n-shaped array from the far-field space, the incident directions are (15°,55°), (25°,60°), (30°,20°), (45°,30°) respectively. The numbers of array elements in the three subarrays of the n-shaped array, namely the *x*-axis subarray, the *y*-axis subarray, and the subarray parallel to the *x*-axis, are all set to 8, while the other parameters remain the same as above. The estimation results are shown in Figure 7. As can be observed from Figure 7, the estimated angles are generally consistent with the true values, indicating that the proposed algorithm remains effective when the number of sources is greater than two.

Simulation Experiment 2: For coherent signals, the effectiveness of the proposed improved MUSIC-based near-field sound source localization algorithm is validated using an n-shaped array.

**Figure 8:**
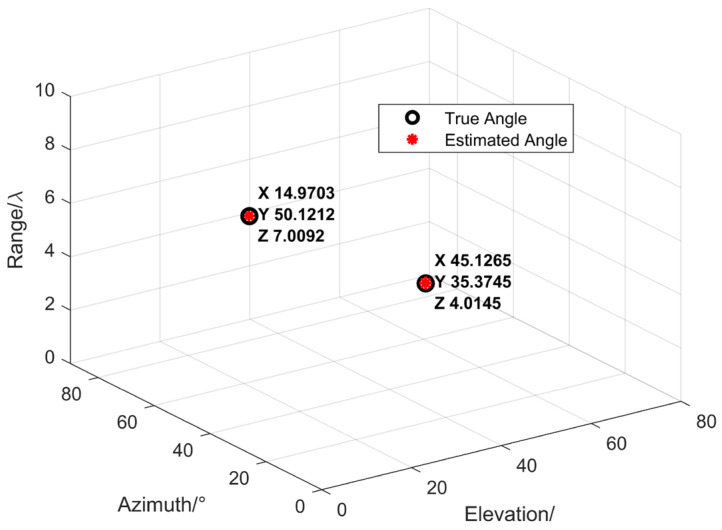
Estimation results of the near-field acoustic source localization algorithm for *K* = 2.

Two narrowband coherent acoustic sources impinge on an n-shaped array, denoted by $s_{1}$ and $s_{2}$. The incident angles and ranges of the sources are characterized by the parameter set (θ,φ,r), where θ denotes the elevation angle, φ denotes the azimuth angle, and r denotes the range. The incident directions of the two sources are specified by (45°,35°,4λ) and (15°,50°,8λ), respectively. The n-shaped array consists of three subarrays aligned along the *x*-axis, the *y*-axis, and parallel to the *x*-axis, each containing M=9 array elements. The sound speed is denoted by c=1500 m/s, and the inter-element spacing is d=λ/4. SNR=20 dB, and the number of snapshots is 500. The estimation results are illustrated in Figure 8. It can be observed that the estimated angles are in good agreement with the true values, demonstrating that the proposed improved MUSIC-based near-field sound source localization algorithm based on the n-shaped array is effective and achieves satisfactory estimation accuracy.

**Figure 9:**
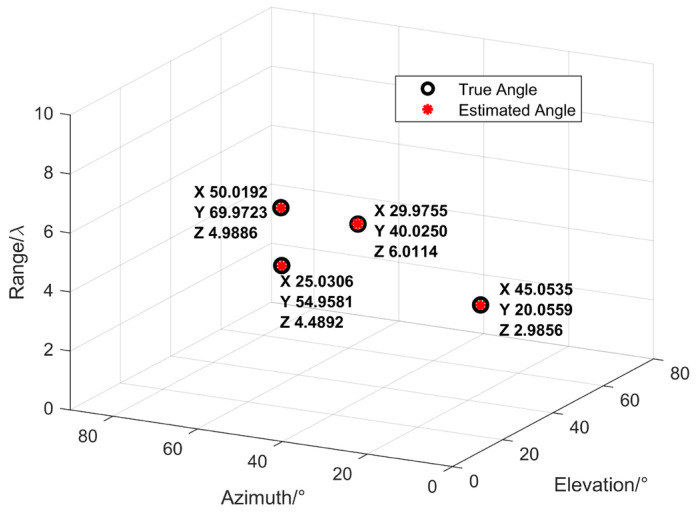
Estimation results of the near-field acoustic source localization algorithm for *K* = 4.

When K=4 narrowband coherent sources impinge on the n-shaped array from the near-field space, the incident directions are (30°,40°,6λ), (45°,20°,3λ), (25°,55°,4.5λ), and (50°,15°,5λ), respectively. The numbers of array elements in the three subarrays of the n-shaped array, namely the *x*-axis subarray, the *y*-axis subarray, and the subarray parallel to the *x*-axis, are all set to 10, while the other parameters remain the same as above. The estimation results are shown in Figure 9. As can be observed from Figure 9, the estimated angles are generally consistent with the true values, indicating that the proposed algorithm remains effective when the number of sources is greater than two.

### 4.2. Performance Analysis

#### 4.2.1. RMSE Versus SNR

In this paper, the improved CBF algorithm and the improved MVDR algorithm are selected for comparison with the proposed method. The proposed approach is developed on the basis of the MUSIC algorithm in combination with the SAGE algorithm and represents a high-resolution subspace-based estimation method. The improved CBF algorithm represents conventional spatial spectrum beamforming methods, whereas the improved MVDR algorithm represents adaptive beamforming methods with stronger interference suppression capability. By comparing these three categories of methods under identical array configurations and signal conditions, a relatively representative performance evaluation framework can be established, which facilitates a clearer assessment of the performance improvement achieved by the proposed algorithm. The following presents the simulation experiments.

Simulation Experiment 3: RMSE versus SNR of angle estimation for the improved MUSIC-based far-field sound source localization algorithm, the improved CBF, and the improved MVDR algorithm.

**Figure 10:**
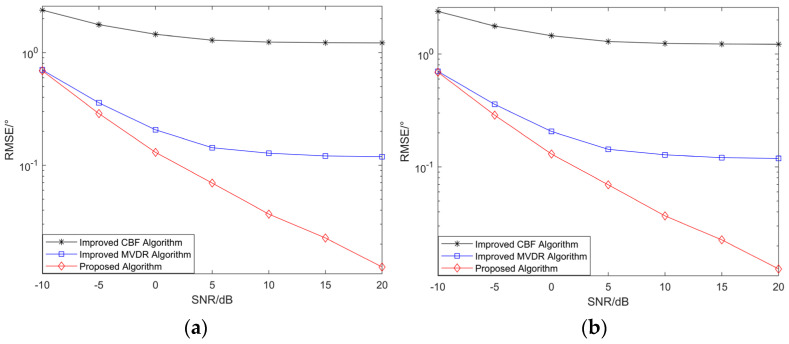
(**a**) RMSE of elevation angle versus SNR; (**b**) RMSE of azimuth angle versus SNR.

Two narrowband coherent sound sources, denoted by $s_{1}$ and $s_{2}$, impinge on the n-shaped array, with their incident directions given by (45.34°,30.12°) and (25.54°,60.25°), respectively. The number of snapshots is set to 500, and the SNR ranges from −10 dB to 20 dB with a step size of 5 dB. For each SNR level, 200 Monte Carlo trials are conducted. Other parameters are the same as those used in Simulation Experiment 1. Figure 10 presents the curves of the RMSE of the elevation and azimuth angle estimates versus the SNR for the three algorithms.

As shown in Figure 10, as the SNR increases, the RMSE of the angle estimates for all three algorithms gradually decreases, indicating that reduced noise influence leads to improved estimation accuracy. Under coherent signal conditions, the proposed improved MUSIC-based far-field sound source localization algorithm consistently achieves the smallest RMSE across all SNR levels, demonstrating superior angle estimation performance compared with the improved MVDR [4,14] and CBF [31] algorithms. The proposed algorithm also maintains good performance under low-SNR conditions; when the SNR is −5 dB, the RMSEs of the elevation and azimuth angles are approximately 0.3° and 0.5°, respectively, demonstrating the robustness of the algorithm.

Simulation Experiment 4: RMSE versus SNR for the improved MUSIC-based near-field sound source localization algorithm, the improved CBF algorithm, and the improved MVDR algorithm.

**Figure 11:**
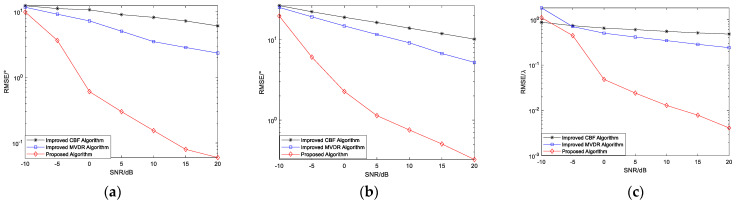
(**a**) RMSE of elevation angle versus SNR; (**b**) RMSE of azimuth angle versus SNR; (**c**) RMSE of range versus SNR.

Two narrowband coherent sound sources, denoted by $s_{1}$ and $s_{2}$, impinge on the n-shaped array, with their incident angles and ranges given (45°,35°,4λ) and (15°,50°,8λ), respectively. The number of snapshots is set to 500, and the SNR ranges from −10 dB to 20 dB with a step size of 5 dB. For each SNR level, 200 Monte Carlo trials are conducted. Other parameters are the same as those used in Simulation Experiment 2. Figure 11 presents the curves of the RMSE of the elevation angle, azimuth angle, and range estimates versus the SNR for the three algorithms.

As shown in Figure 11, the proposed algorithm achieves higher estimation accuracy for all three parameters than the improved MVDR and CBF algorithms, and the RMSE of both the angle and range estimates decreases gradually as the SNR increases. Under low-SNR conditions, noise components affect the statistical characteristics of the covariance matrix, thereby degrading parameter estimation accuracy and resulting in relatively large errors. As the SNR increases, the influence of noise diminishes, thereby improving the parameter estimation accuracy. The proposed algorithm applies spatial smoothing to the subarray data and constructs an equivalent covariance matrix to restore the rank of the signal subspace under coherent-source conditions, thereby avoiding estimation errors caused by rank deficiency. By combining coarse estimation using MUSIC with refined optimization using the SAGE algorithm, the method reduces discretization errors and the impact of parameter coupling, enabling the rapid and stable estimation of angle and range parameters under medium- to high-SNR conditions. As a result, the RMSE performance and stability of the proposed algorithm are consistently superior to those of the comparison algorithms, demonstrating its robustness and high-precision estimation capability.

#### 4.2.2. RMSE Versus Number of Snapshots

Simulation Experiment 5: RMSE of angle estimation versus the number of snapshots for the improved MUSIC-based far-field sound source localization algorithm, the improved CBF algorithm, and the improved MVDR algorithm

**Figure 12:**
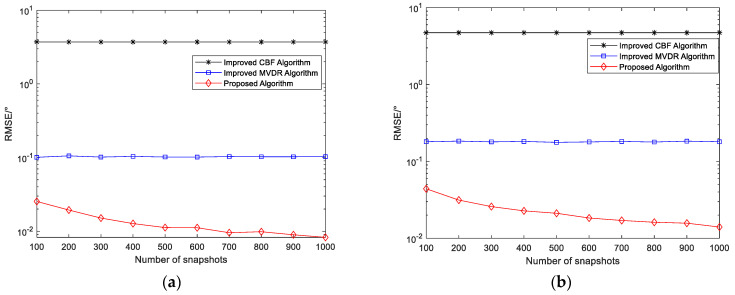
(**a**) RMSE of elevation angle versus number of snapshots; (**b**) RMSE of azimuth angle versus number of snapshots.

Two narrowband coherent sound sources, denoted by $s_{1}$ and $s_{2}$, impinge on the n-shaped array, with their incident directions given by (45.34°,30.12°) and (25.54°,60.25°), respectively. SNR=20 dB. The number of snapshots varies from 100 to 1000, and 200 Monte Carlo trials are conducted for each snapshot setting. The other parameters are the same as those used in Simulation Experiment 1. Figure 12 presents the curves of the RMSE of the elevation and azimuth angle estimates versus the number of snapshots for the three algorithms.

As shown in Figure 12, as the number of snapshots increases, the RMSE of the angle estimates obtained by the proposed algorithm gradually decreases, indicating that the method effectively exploits the additional statistical information provided by more snapshots, resulting in improved subspace estimation stability and enhanced parameter estimation accuracy. In contrast, the RMSE of the improved MVDR and CBF algorithms decreases only marginally as the number of snapshots increases and tends to saturate, primarily because these algorithms are limited by their resolution and interference suppression capabilities. With a small number of snapshots, the RMSE of the proposed algorithm is relatively large, mainly due to the presence of coherent signals, as a limited sample size reduces the accuracy of covariance matrix estimation, leading to degraded separation between the signal and noise subspaces and consequently increased parameter estimation errors. However, as the number of snapshots increases, this effect diminishes rapidly, and the RMSE curve stabilizes while maintaining a lower error level, further validating the advantages of the proposed method in terms of statistical stability and estimation accuracy.

Simulation Experiment 6: RMSE values of angle and estimation versus the number of snapshots for the improved MUSIC-based near-field sound source localization algorithm, the improved CBF algorithm, and the improved MVDR algorithm

**Figure 13:**
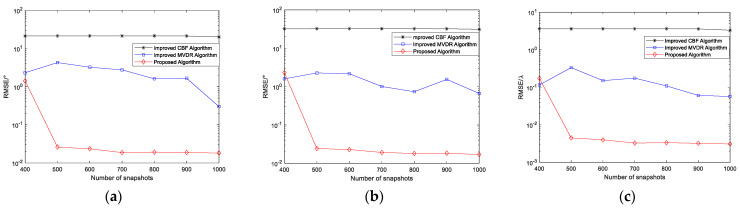
(**a**) RMSE of elevation angle versus number of snapshots; (**b**) RMSE of azimuth angle versus number of snapshots; (**c**) RMSE of range versus number of snapshots.

Two narrowband coherent sound sources, denoted by $s_{1}$ and $s_{2}$, impinge on the n−shaped array, with their incident angles and ranges given by (45.34°,30.12°) and (25.54°,60.25°), respectively. SNR=20 dB. The number of snapshots varies from 400 to 1000, and 200 Monte Carlo trials are conducted for each snapshot setting. The other parameters are the same as those used in Simulation Experiment 2. Figure 13 presents the curves of the RMSE of the elevation angle, azimuth angle, and range estimates versus the number of snapshots for the three algorithms.

Under coherent signal conditions, Figure 13 shows the RMSE of the elevation angle, azimuth angle, and range estimates as functions of the number of snapshots, respectively. The results show that the proposed algorithm achieves superior estimation performance for all three parameters compared with the improved CBF and MVDR algorithms. When the number of snapshots increases from 400 to 500, the RMSE of the proposed algorithm decreases significantly, primarily because, in the presence of coherent signals, a limited sample size reduces the accuracy of covariance matrix estimation, resulting in degraded separation between the signal and noise subspaces. As the number of snapshots increases, the sample statistics gradually converge, the subspace estimation error diminishes rapidly, and the spatial spectrum becomes more stable, resulting in a significant reduction in RMSE. This behavior demonstrates the good convergence properties and robustness of the proposed algorithm.

#### 4.2.3. Comparison of Different Array Configurations

Simulation Experiment 7: RMSE versus SNR for coherent signals using an improved MUSIC-based far-field sound source localization algorithm with n-shaped and L-shaped arrays.

**Figure 14:**
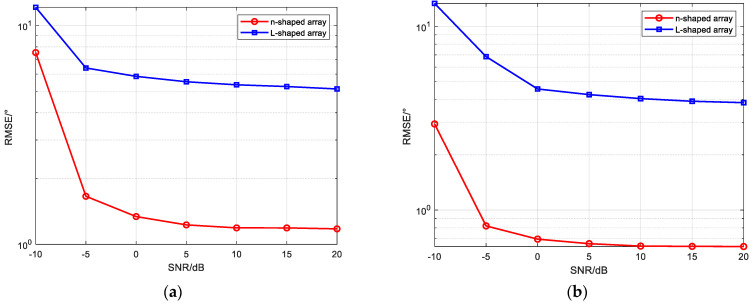
(**a**) RMSE of elevation angle versus number of SNR; (**b**) RMSE of azimuth angle versus number of SNR.

The structure of the n-shaped array is shown in Figure 2, where each of the three subarrays consists of eight elements. The L-shaped array is composed of two subarrays aligned along the *x*-axis and the *y*-axis, each containing eight elements. The inter-element spacing of both arrays is set to d=λ/2. The number of snapshots is 200, and the SNR varies from −10 dB to 20 dB with a step size of 5 dB. Two narrowband coherent sound sources impinge on the n-shaped array and L-shaped array, respectively, with identical incident angles and ranges. For each SNR level, 200 Monte Carlo trials are performed. Figure 14 shows the RMSE curves of the elevation and azimuth angle estimates versus the SNR for far-field sound source localization using the improved MUSIC algorithm based on the two array configurations.

As shown in Figure 14, the n-shaped array shows relatively large estimation errors at −10 dB; however, when the SNR increases from −10 dB to −5 dB, the RMSE decreases significantly. As the SNR increases further, the RMSE gradually stabilizes. This behavior can be attributed to the reduced influence of noise on the array covariance matrix at higher SNR levels, resulting in a corresponding decrease in estimation error.

Across all SNR conditions, the RMSEs of both the elevation and azimuth angle estimates obtained using the n-shaped array are smaller than those obtained with the L-shaped array. This can be attributed to the more balanced element distribution of the n-shaped array in two-dimensional space, which provides a more effective array aperture and enables the proposed algorithm to achieve lower estimation errors when implemented with this configuration. In contrast, the two-dimensional spatial sampling structure of the L-shaped array is more limited and exhibits lower sensitivity to angular variations. In the presence of noise perturbations, the main peak of the spatial spectrum becomes more susceptible to shifts, thereby reducing peak localization accuracy and increasing parameter estimation errors.

Simulation Experiment 8: RMSE versus SNR for coherent signals using an improved MUSIC-based near-field sound source localization algorithm with n-shaped and L-shaped arrays.

**Figure 15:**
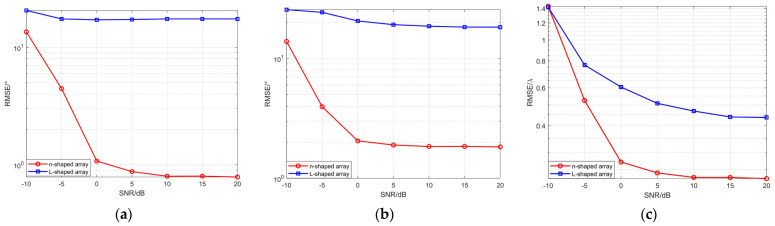
(**a**) RMSE of elevation angle versus SNR; (**b**) RMSE of azimuth angle versus SNR; (**c**) RMSE of range versus SNR.

Each of the three subarrays of the n-shaped array consists of M=7 elements, while each subarray of the L-shaped array contains L=10 elements. The two array configurations have the same total number of array elements, which is 19, and identical inter-element spacing is set to d=λ/4. The number of snapshots is set to 200, and SNR varies from −10 dB to 20 dB with a step size of 5 dB. Two narrowband coherent sound sources impinge on the n-shaped array and the L-shaped array, respectively, with identical incident angles and identical ranges. For each SNR level, 200 Monte Carlo trials are performed. Figure 15 presents the RMSE curves of the angle and range estimates versus SNR for the two array configurations, obtained using the improved MUSIC-based near-field sound source localization algorithm.

As shown in Figure 15, the n-shaped array exhibits relatively large estimation errors for the target parameters under low SNR conditions; however, as the SNR increases, the influence of noise on the sample covariance matrix diminishes, leading to more accurate subspace estimation and a corresponding significant reduction in parameter estimation errors. With an equal total number of array elements, the application of the proposed near-field sound source localization algorithm enables the n-shaped array to achieve lower RMSEs in the estimation of elevation, azimuth, and range parameters, while exhibiting a decreasing and more stable error trend as the SNR increases.

The simulation results demonstrate the advantage of the n-shaped array in joint angle and range estimation. By employing a multi-directional element layout to form a larger array aperture, the n-shaped array improves steering vector diversity and subspace stability, thereby enhancing parameter resolution and noise robustness, which enables lower estimation errors under different SNR conditions. In contrast, the geometric degrees of freedom of the L-shaped array are more limited, restricting its spatial sampling capability; consequently, it exhibits higher RMSEs across the entire SNR range.

## 5. Conclusions

To meet the requirements of long-range direction estimation and short-range precise localization in the AUV localization process, this paper presents far-field and near-field acoustic source localization algorithms based on the n-shaped array. The proposed method first constructs a spatially smoothed data vector and subsequently forms the corresponding equivalent covariance matrix. The MUSIC algorithm is then employed to obtain coarse angle estimates, which serve as initializations for the SAGE algorithm. The SAGE algorithm is subsequently employed to refine the angular and range parameters, thereby enhancing estimation accuracy. Simulation results demonstrate that, under both far-field and near-field conditions and in the presence of coherent sources, the proposed algorithm outperforms the improved CBF and MVDR algorithms, yielding lower estimation errors and superior estimation accuracy. Furthermore, when the proposed algorithm is applied to compare the n-shaped array with the commonly used two-dimensional L-shaped array, the n-shaped array achieves superior estimation performance, thereby demonstrating the effectiveness of the n-shaped array design.

In addition, although the proposed n-shaped array–based far-field and near-field acoustic source localization method demonstrates promising performance in simulation studies, practical underwater acoustic environments are typically characterized by significant complexity and uncertainty. Factors such as environmental mismatch and colored noise may influence both the array signal model and the performance of the algorithm. In practical engineering applications, mechanical installation errors and platform motion may cause the array geometry to deviate from the ideal array model. Small position deviations of the hydrophones may lead to steering vector mismatch, thereby degrading parameter estimation accuracy. Furthermore, in practical applications, the number of sources is often difficult to determine accurately in advance, which places higher demands on the robustness of the algorithm. In this work, the performance characteristics of the proposed algorithm are mainly analyzed under controlled simulation conditions, while the effects of environmental uncertainties, array geometry mismatches, and platform motion on localization performance, as well as the corresponding compensation strategies, will be further investigated and validated in future studies.

Future research will focus on the following aspects. First, underwater experiments will be conducted to further investigate the effects of environmental uncertainties in complex underwater conditions, array geometry mismatches, and platform motion on localization performance, to evaluate the engineering feasibility and operational stability of the proposed algorithm. Second, when the number of sources is unknown, information-theoretic criteria such as the Akaike Information Criterion (AIC) or the Minimum Description Length (MDL) criterion can be incorporated into the proposed algorithm to improve its applicability in complex practical environments. In addition, for parameter estimation under limited snapshot or low SNR conditions, the robustness and computational efficiency of the algorithm can be further enhanced. Finally, the potential application of the n-shaped array configuration in other array signal processing problems may also be investigated. These research directions will contribute to further improving the algorithmic performance and its practical engineering applicability.

## Figures and Tables

**Table 1:** Literature summary.

Reference	Array Geometry	Signal Model	Signal Characteristics	Location Method
[14]	ULA	near-field	non-coherent	MVDR
[17]	planar array	near-field	non-coherent	BA, MUSIC
[19]	L-shaped array	far-field	coherent	SS, ESPRIT
[20]	ULA	far-field	coherent	Matrix Reconstruction MUSIC
[21]	ULA	far-field	coherent	Matrix Reconstruction ESPRIT
[23]	ULA	near-field	coherent	AOP, ML
[25]	planar array	near-field	coherent	Covariance Matrix Construction, MUSIC

## Data Availability

Data is contained within the article.

## Abbreviations

The following abbreviations are used in this article:

AUV	Autonomous underwater vehicle
MUSIC	Multiple Signal Classification
SAGE	Space-Alternating Generalized Expectation-maximization
ULA	Uniform Linear Array
DOA	Direction of arrival
CBF	Conventional beamforming
MVDR	Minimum Variance Distortionless Response
ESPRIT	Estimation of Signal Parameters via Rotational Invariance Technique
PM	Propagator method
ML	Maximum likelihood
CA	Covariance approximation
AOP	Alternating oblique projection
TR	Time-reversal
SVD	singular value decomposition
RMSE	Root mean square error
SNR	Signal-to-noise ratio
BA	Bat algorithm
SS	Spatial Smoothing
E-step	Expectation step
M-step	Maximization step
AIC	Akaike Information Criterion
MDL	Minimum Description Length
